# Characterizing the diversity and commensal origins of *penA* mosaicism in the genus *Neisseria*

**DOI:** 10.1099/mgen.0.001209

**Published:** 2024-02-21

**Authors:** Anastasia Unitt, Martin Maiden, Odile Harrison

**Affiliations:** ^1^​ Department of Biology, University of Oxford, Oxford, OX1 3SY, UK; ^2^​ Infectious Disease Epidemiology Unit, Nuffield Department of Population Health, University of Oxford, OX3 7LF, UK

**Keywords:** *Neisseria gonorrhoeae*, genomics, antimicrobial resistance, mosaic, penicillin binding protein 2, *penA*, commensals

## Abstract

Mosaic *penA* alleles formed through horizontal gene transfer (HGT) have been instrumental to the rising incidence of ceftriaxone-resistant gonococcal infections. Although interspecies HGT of regions of the *penA* gene between *Neisseria gonorrhoeae* and commensal *Neisseria* species has been described, knowledge concerning which species are the most common contributors to mosaic *penA* alleles is limited, with most studies examining only a small number of alleles. Here, we investigated the origins of recombinant *penA* alleles through *in silico* analyses that incorporated 1700 *penA* alleles from 35 513 *Neisseria* isolates, comprising 15 different *Neisseria* species. We identified *Neisseria subflava* and *Neisseria cinerea* as the most common source of recombinant sequences in *N. gonorrhoeae penA*. This contrasted with *Neisseria meningitidis penA*, for which the primary source of recombinant DNA was other meningococci, followed by *Neisseria lactamica*. Additionally, we described the distribution of polymorphisms implicated in antimicrobial resistance in *penA*, and found that these are present across the genus. These results provide insight into resistance-related changes in the *penA* gene across human-associated *Neisseria* species, illustrating the importance of genomic surveillance of not only the pathogenic *Neisseria*, but also of the oral niche-associated commensals from which these pathogens are sourcing key genetic variation.

## Data Summary

Nucleotide sequence data were extracted from the PubMLST database (https://pubmlst.org/neisseria), which hosts publicly available bacterial genetic data from various sources. Table S1, available in the online version of this article, describes accession numbers and references for each isolate used. In addition to this the NG-STAR database was used as a further source of *penA* alleles (https://ngstar.canada.ca/pages/eula?lang=en).

**Impact Statement**

The literature concerning the *penA* gene, which encodes penicillin binding protein 2 in the genus *Neisseria*, has described the involvement of mosaic *penA* variants in the generation of reduced susceptibility and resistance towards extended-spectrum cephalosporins (ESCs), and characterized key polymorphisms within them that contribute to this phenotype. Here we combine this information with an analysis of the *penA* gene in genomes from over 35 000 isolates representing 15 *Neisseria* species, providing insight into the characteristics of this resistance determinant across the genus. This dataset enabled us to assess the origins of mosaic *penA* alleles through recombination analyses, identifying the commensals responsible for the horizontal gene transfer of common antimicrobial resistance-associated regions to the pathogenic *Neisseria* species *N. meningitidis* and *N. gonorrhoeae*. These findings illustrate the importance of certain commensal species as reservoirs of polymorphisms that contribute to reduced susceptibility to antimicrobials in this highly recombinogenic genus*,* and provide an assessment of the population genetics of the *penA* gene in the genus *Neisseria* up to the year 2022. These data can be applied to inform efforts aiming to control and counteract the evolution of resistance to ESCs.

## Introduction

The WHO 2018 report on global sexually transmitted infection surveillance characterizes gonorrhoea as a highly prevalent sexually transmitted infection, second only to chlamydia, with antimicrobial resistance (AMR) a growing public health concern in circulating gonococci [1]. Since the first instances of reduced susceptibility to antimicrobials in the mid-1900s [2], *N. gonorrhoeae* has gone on to develop resistance to many classes of antibiotics, with the extended-spectrum cephalosporin (ESC), ceftriaxone, representing the last monotherapy available for the effective clearance of disease [3]. Reduced susceptibility to ESCs can arise in gonococci through spontaneous mutations or horizontal gene transfer (HGT) of both chromosomal and plasmid-based resistance determinants [2]. Members of the genus *Neisseria* are highly recombinogenic, and, as such, the horizontal transfer of polymorphisms by transformation represents an important mechanism through which *N. gonorrhoeae* can acquire and disseminate genetic sequences that reduce its susceptibility to antimicrobials [4, 5].

The *penA* gene, which encodes penicillin binding protein 2 (PBP2), exemplifies the role of HGT in the generation of AMR. In the 1990s, mosaic forms of this gene were identified in gonococci, and shown to confer reduced susceptibility to ESCs [6–8]. These variants were described as mosaic due to their patchwork of sequences of different genetic origins, derived through HGT and intragenic recombination of subsections of the gene from other strains, and other *Neisseria* species [8, 9]. Mosaic regions in gonococcal *penA* alleles have been observed to show homology with *penA* sequences present in species such as *Neisseria lactamica, Neisseria cinerea*, *Neisseria subflava* and *Neisseria meningitidis* [8, 10]. The mosaic region is usually found in the latter half of the *penA* gene [8].

Resistance to ESCs involves multiple genes and evolves in a cumulative manner [11, 12]. However, phylogenetic and transformation studies have shown that mosaic variants of the *penA* gene are responsible for the majority of reduced susceptibility to ESCs in gonococci [11, 13]. Specific polymorphisms within *penA* have been identified as being critical to the generation of resistance by mosaic alleles, particularly substitutions resulting in the following amino acid changes: I312M, V316T, A501V, N513Y and G546S [14–16]; however, these polymorphisms alone are not sufficient to generate the full resistance phenotype produced by mosaic *penA* alleles, due to epistatic effects [12, 17].

Mosaic *penA* alleles are categorized into distinct mosaic types, based on an 83 amino acid nomenclature which spans positions 35–576 in the *penA* gene [18]. Certain *penA* mosaic types have been identified as inducing particularly strong resistance to ESCs, specifically types 10 and 34 (also known as type X or XXXIV, although the roman number system has become cumbersome as more types are characterized) [18–20]. These types are used in AMR typing schemes such as NG STAR, which includes *penA* allele/type as a predictor of resistance phenotype [19]. However, despite the consistent characterisation of new mosaic *penA* types, the source of mosaic regions (although regularly referred to as originating from commensal *Neisseria*) lacks a systemic analysis in the literature.

The widespread availability of bacterial genome sequence data presents an opportunity to explore AMR determinants at the population level, providing insight into how processes such as HGT influence the development and dissemination of such determinants. In this study, a dataset of 1700 *penA* alleles from 15 *Neisseria* species was investigated. Through alignment, clustering and recombination analysis, we characterized the diversity of *penA* alleles, and identified that some commensal species contribute more to the current population of recombinant *penA* alleles than others.

## Methods

### Dataset selection

The *Neisseria* PubMLST database (https://www.pubmlst.org/Neisseria) was used to download a total of 1700 *penA* alleles. Within the *Neisseria* database the *penA* locus is known as NEIS1753. The species from which these alleles were derived was determined, with *Neisseria* species identity confirmed using rMLST (ribosomal multi-locus sequence typing) [21]. Only human-associated *Neisseria* were included, consisting of 15 species: ‘*N. benedictiae’, N. bergeri, ‘N. blantyrii’, N. cinerea, N. elongata, N. gonorrhoeae, N. lactamica, ‘N. maigaei’, N. meningitidis, N. mucosa, N. oralis, N. polysaccharea, N. subflava, ‘N. uirgultaei’ and ‘N. viridiae’* (Table S1). Alleles from isolates with >400 contigs were excluded, as this was used as an indication of poor-quality draft genome data which could impact downstream analyses.

### Sequence and recombination analyses

Multiple sequence alignments were generated using both nucleotide and deduced amino acid sequences, with Muscle 3.8.425 in mega V10 [22]. These included 1700 *penA* allele sequences from PubMLST. The ‘*Neisseria gonorrhoeae* Sequence Typing for Antimicrobial Resistance’ (NG-STAR) typing scheme utilizes *penA* as one of its seven gonococcal AMR genes, and the accompanying NG-STAR website (https://ngstar.canada.ca/) hosts a collection of known mosaic *penA* alleles from *N. gonorrhoeae* [23]. The definition of mosaic types in NG-STAR is based on an 83 amino acid nomenclature previously described by Ohnishi *et al*. [18]. Each *penA* type may include multiple *penA* alleles; for example, there are multiple *penA* alleles which contain the mosaic 34 motif (Fig. S2) associated with resistance to ESCs, each possessing amino acid polymorphisms specific to mosaic 34 but including additional synonymous and non-synonymous changes in other regions of the gene [24]. In addition to mosaic types, NG-STAR also annotates semi-mosaic *penA* types, where the mosaic region only covers one of the two key regions of the *penA* gene defined in the original 83 amino acid *penA* mosaic type nomenclature [23]. Types defined in NG-STAR correspond with certain alleles in PubMLST, such that, for example, NEIS1753 allele 266 corresponds with NG-STAR *penA* 34.001, NEIS1753 allele 1240 matches NG-STAR *penA* allele 34.002 and so on (Table S1).

Recombination analyses were performed using RDP5, a recombination detection program which identifies recombination events from nucleotide sequence alignments, based on a combination of multiple recombination detection tools and statistics [25]. As part of its output, RDP5 identifies minor parents: alleles predicted to have contributed the smaller fraction of a recombinant allele, and major parents: alleles predicted to have provided the genetic background into which the recombinant region was transferred. Counts of how often particular species were identified as donating the minor parental allele of a recombinant sequence provided insight into the patterns of contribution to recombinant *penA* alleles.

Recombination analyses were performed on a curated, representative sub-dataset consisting of 1061 *penA* nucleotide sequences, generated to conform with the requirements specified by RDP5 [25]. This included the removal of 638 highly conserved *N. meningitidis penA* alleles, as well as a highly divergent *N. elongata* allele, so as to reduce the possibility of false recombination events being detected (Table S1).

Neighbour joining trees were generated to visualize the clustering of *penA* alleles using mega and FastTree 2.1.1 using default parameters [22, 26]. These trees were then edited and annotated using ITOL [27].

### Characterization of *penA* amino acid substitutions known to confer reduced susceptibility

The following amino acid substitutions at residues I312M, V316T, N513Y, G546S and A502V have been associated with reduced susceptibility to antimicrobials [14, 16, 28]. Amino acid sequences found at these residues were extracted from all 1700 *penA* alleles across all 15 *Neisseria* species, allowing the distribution of AMR-conferring substitutions to be examined across the genus. A concatenated sequence consisting of these amino acid polymorphisms was used to delineate the different variants found. For example, IVANG would represent the combination 312I–316V–502A–513N–546G (associated with susceptibility) and MTVYS 312M–316T–502V–513Y–546S (AMR-associated).

### Statistical analysis and visualization

Shannon’s index of diversity was calculated in R 4.1.2. using vegan version 2.6-4 [29]. Tajima’s D, *p-*distances and other additional diversity indices were calculated using mega x [22]. The python package Cogent3 [30] was employed to determine pairwise distances under the Jukes and Cantor 69 (JC69) substitution model in species for which there were >10 or more alleles. JC69 values were then visualized through violin plots generated using the seaborn python package to allow the range of diversity present within species to be observed [31].

To investigate the relationship between species and mosaic *penA* types, principal component analyses (PCAs) were undertaken. A numeric matrix of amino acid alignments was generated and standardized using the StandardScaler tool in the python package scikit-learn [32]. Given the large matrix, standardization ensured data were stable, and less influenced by the range of variables present. A two-component PCA was then undertaken using the sklearn.decomposition function in scikit-learn. The resulting data were plotted using the pyplot module in matplotlib and visualized by species and mosaic types [33]. Additional data visualization was undertaken using the R package ggplot2 [34].

## Results

### Isolate collection

The assembled dataset included 35 513 isolates belonging to 15 different *Neisseria* species (Table S1). The majority were *N. meningitidis* (22 809, 64.23 %) and *N. gonorrhoeae* (11 933, 33.60 %), with the remainder (771, 2.17 %) from human-associated commensal *Neisseria* species. These included *N. lactamica* (518, 1.46 %), *N. bergeri* (58, 0.16 %), *N. polysaccharea* (53, 0.15 %), *N. subflava* (41, 0.12 %), *N. cinerea* (31, 0.09 %), *N. viridiae* (21, 0.06 %), *N. mucosa* (15, 0.04 %), *N. blantyrii* (14, 0.04 %), *N. uirgultaei* (9, 0.03 %), *N. oralis* (4, 0.01 %), *N. benedictiae* (3, 0.01 %), *N. elongata* (3, 0.01 %) and *N. maigaei* (1, 0.00 %).

Of those isolates with a continent of isolation listed (*n*=35 030), the majority were from Europe (22 096, 63.08 %), followed by North America (5458, 15.58 %). However, historically under-represented regions were also present in this dataset, including: Africa (3626, 10.35 %), Asia (2027, 5.79), Oceania (1186, 3.39 %) and South America (637, 1.82 %). Isolates originated from as early as 1915, but the majority of isolates were collected post-2000, with collection peaking in 2015 (4477).

### Genetic diversity indices for *penA* (NEIS1753)

A total of 1700 *penA* alleles were identified. The majority belonged to *N. meningitidis* (1173, 68.68 %) followed by *N. gonorrhoeae* (216, 12.65 %) and *N. lactamica* (194, 11.36 %). The most common allele was NEIS1753 59, which was identified in 3029 *N*. *meningitidis* isolates characterized as clonal complex ST-11, followed by allele 166 found in 2445 *N*. *gonorrhoeae,* 1058 of which belonged to ST-9363, and allele 8 found in 1935 *N*. *meningitidis* ST-23 clonal complex isolates (Table S1).

The *penA* gene encodes PBP2, an integral part of the peptidoglycan synthesis pathway, and as such all sequences collected were predicted to be functional, although one allele included in the analysis was truncated (allele 2194) and some alleles were highly divergent such as those belonging to *N. elongata*. This is possibly a consequence of the differing cell wall structure found in *N. elongata*; this species is a bacillus, unlike the other species included here, which are diplococci [35].

Shannon’s index of diversity is a measure that combines both the diversity of ‘types’ in a dataset along with the relative abundance of each type. Shannon’s index for *Neisseria penA* alleles by species demonstrated that *penA* diversity was species dependent. For example, *N. meningitidis penA* alleles had a diversity index of 4.15, in comparison to *N. gonorrhoeae penA* alleles with a lower diversity index of 3.06 (Table 1). Some species contributed so few *penA* alleles to the dataset that their diversity index remained at zero or was incalculable.

**Table 1.** Diversity indices for *penA* by species

Species	Shannon’s index of diversity	No. of alleles	Average nucleotide size (bp)	Average peptide size (aa)	No. of segregating sites	Mean *p*-distance	Tajima’s *D*
*Neisseria benedictiae*	0.64	2	1749	583	319	0.22	n/a
*Neisseria bergeri*	1.6	13	1746	582	391	0.09	0.49
*Neisseria blantyrii*	0.51	3	1752	584	337	0.15	n/a
*Neisseria cinerea*	3.18	26	1749	583	364	0.06	−0.13
*Neisseria elongata*	1.1	3	1743–1998	583–586	n/a	n/a	n/a
*Neisseria gonorrhoeae*	3.06	216	1749	583	396	0.04	0.19
*Neisseria lactamica*	3.61	194	1746	582	442	0.04	−0.24
*Neisseria maigaei*	n/a	1	1749	583	n/a	n/a	n/a
*Neisseria meningitidis*	4.15	1173	1746	582	820	0.05	−0.74
*Neisseria mucosa*	2.52	13	1749	583	300	0.06	0.34
*Neisseria oralis*	1.39	4	1746	582	59	0.02	−0.17
*Neisseria polysaccharea*	2.99	28	1749	583	513	0.10	0.76
*Neisseria subflava*	3.23	28	1749	583	297	0.40	−0.50
*Neisseria uirgultaei*	n/a	1	1749	583	n/a	n/a	n/a
*Neisseria viridiae*	0.59	3	1746	582	22	0.00	n/a

Tajima’s *D* was used to determine whether *penA* nucleotide sequences are evolving randomly (*D*=0) or are subject to non-random evolutionary processes such as selective pressure (*D*<0 or *D*>0) [36]. Tajima’s *D* values obtained ranged from −0.24 to 0.76 across the species examined. Negative Tajima’s *D* values were found for *N. cinerea* (−0.13, *n*=26)*, N. lactamica* (−0.24, *n*=194)*, N. meningitidis* (−0.74, *n*=1173)*, N. oralis* (−0.17, *n*=4) and *N. subflava* (−0.5, *n*=28) which indicated a higher incidence of rare alleles [36]. Meanwhile positive Tajima’s *D* values were found for *N. polysaccharea* (0.76, *n*=28), *N. bergeri* (0.49, *n*=13), *N. mucosa* (0.34, *n*=13) and *N. gonorrhoeae* (0.19, *n*=216) suggesting a dearth of rare alleles (Table 1). However all these values were relatively small, being >−1 but <1.

**Fig. 1.**
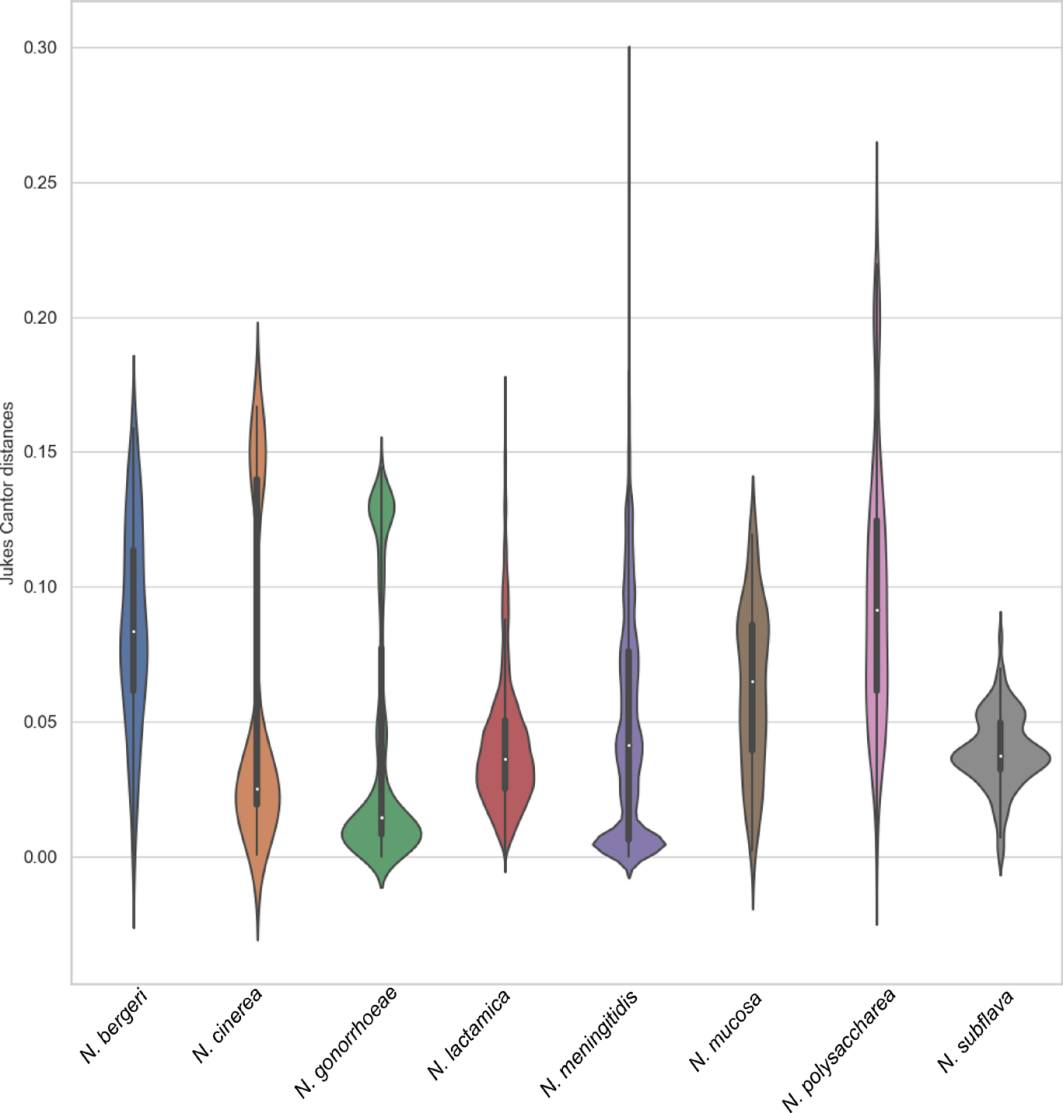
Violin plots denoting *penA* pairwise distances obtained within each *Neisseria* species. Pairwise distance values were obtained under the Jukes–Cantor substitution model and using the python package Cogent3 [30]. Violin plots were then generated using the python package Seaborn [31]. Only species with 10 or more *penA* alleles were included in analyses.

**Fig. 2.**
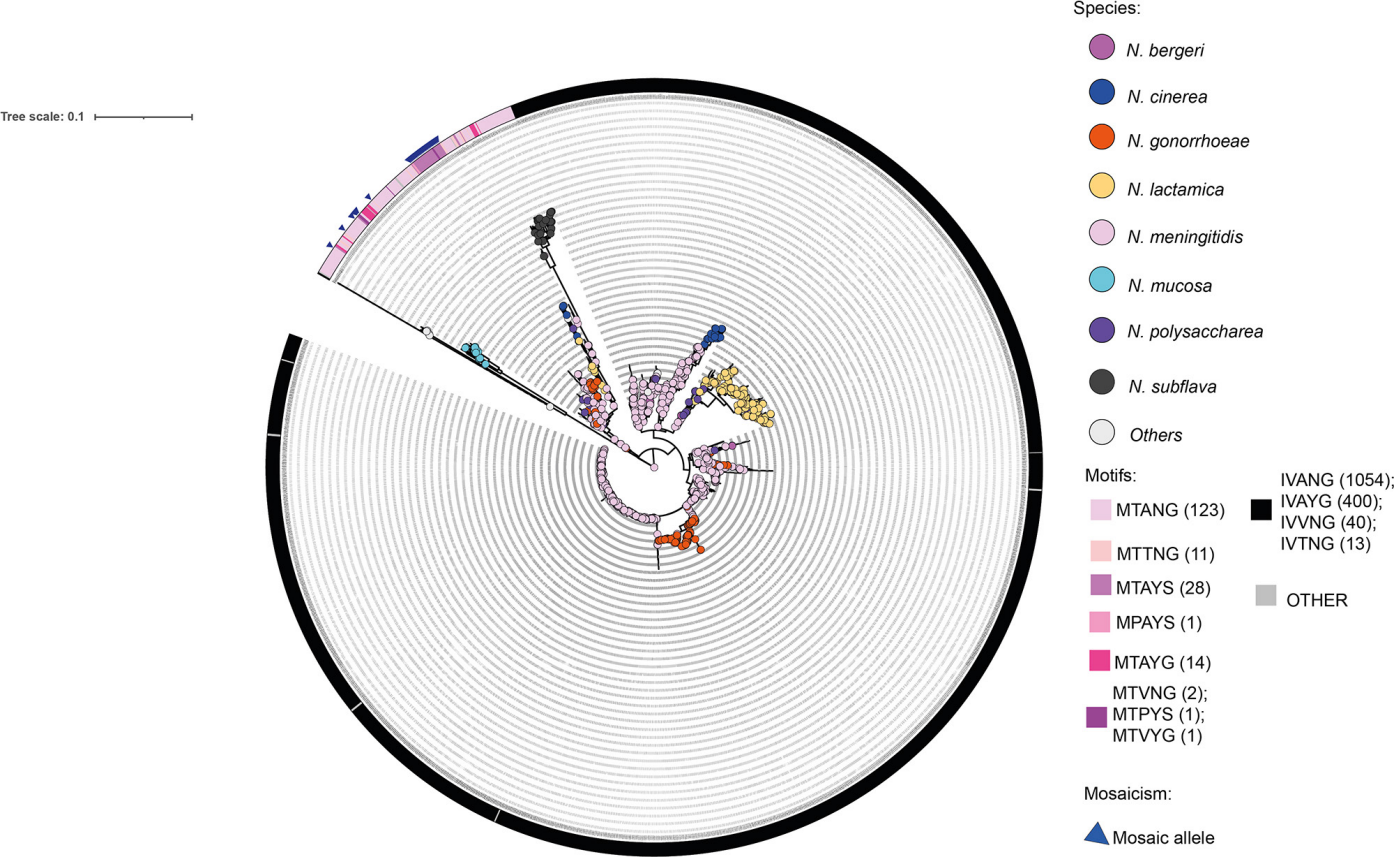
Neighbour-joining tree depicting *penA* alleles belonging to 15 *Neisseria* species. Nucleotide sequence alignments were generated using mega after which a neighbour-joining tree was derived using the Jukes–Cantor model and 100 bootstraps. The resulting newick file was annotated in iTOL [27]. Leaves are annotated by species with *‘N. blantyrii’, ‘N. benedictiae’, N. elongata, ‘N. maigaei’, N. oralis, ‘N. viridiae’ and ‘N. uirgultaei’* grouped together under ‘Others’ given the small number of isolates belonging to these species. The tree was further annotated by motif distribution with motifs associated with AMR highlighted using pink hues. Other motifs were annotated in grey or black. Mosaic *penA* alleles are denoted with blue triangles.

**Fig. 3.**
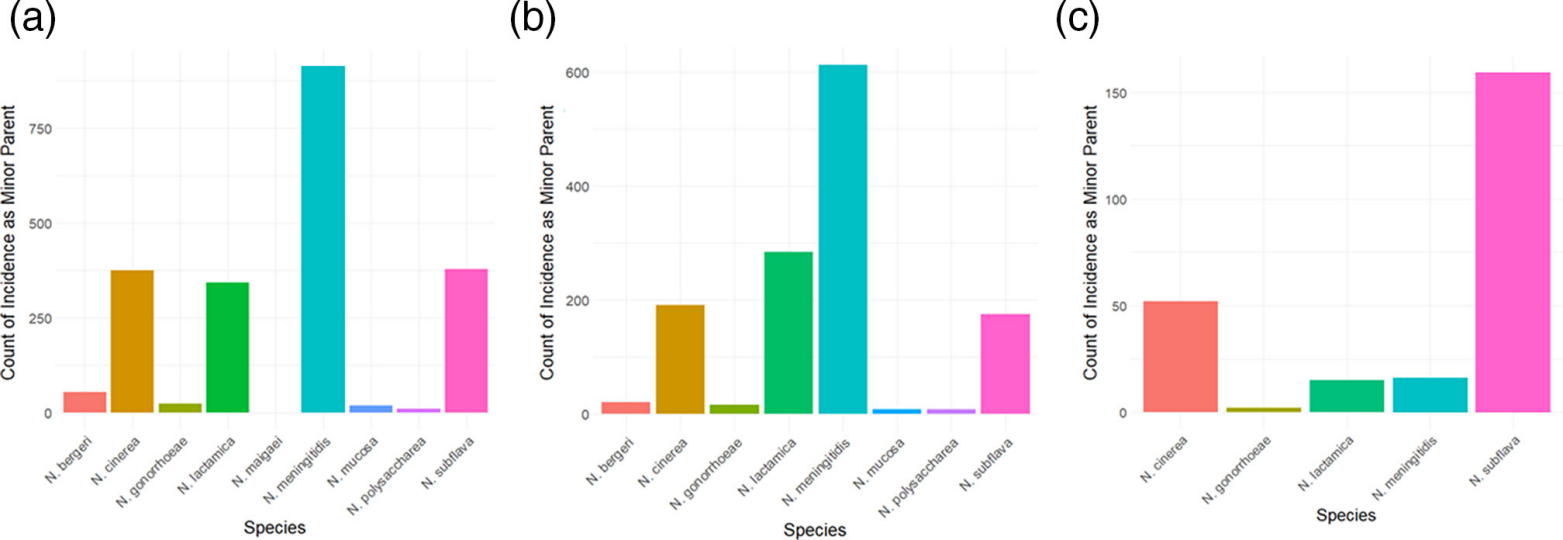
RDP5 analyses and identification of minor parents. (a) Species’ incidence as minor parent of recombinant *penA* alleles across recombination analyses. Recombination analysis performed using RDP5 identified putative recombination events and the minor parental species in these events. Minor parents are alleles predicted to have contributed the smaller fraction of a recombinant allele. Across the 1061 allele analysis, *N. lactamica* was identified as the most common minor parent, followed by *N. cinerea* and *N. mucosa*. (b) Species’ incidence as minor parent in recombinant *N. meningitidis penA* alleles. In recombinant alleles belonging to *N. meningitidis,* intraspecies recombination from other *N. meningitidis* alleles was the most common minor parent. (c) Species’ incidence as minor parent in recombinant *N. gonorrhoeae penA* alleles. In *N. gonorrhoeae* recombinant alleles, *N. subflava* was the most common minor parent.

Pairwise distance values, obtained using the Jukes–Cantor substitution model, identified distinct populations of *penA* alleles in *N. cinerea, N. gonorrhoeae* and *N. meningitidis* evidenced by the presence two or more clusters in the violin plots (Fig. 1). Thus, although the mean distance obtained represents the overall diversity found within each species, examining all distance indices identified outliers and other more discrete allelic populations that may be present. These data are consistent with the observation of divergent *N. cinerea, N. meningitidis* and *N. gonorrhoeae penA* alleles revealed in subsequent phylogenetic and PCA analyses (Figs 2 and 3).

### Neighbour-joining trees and PCAs

**Fig. 4.**
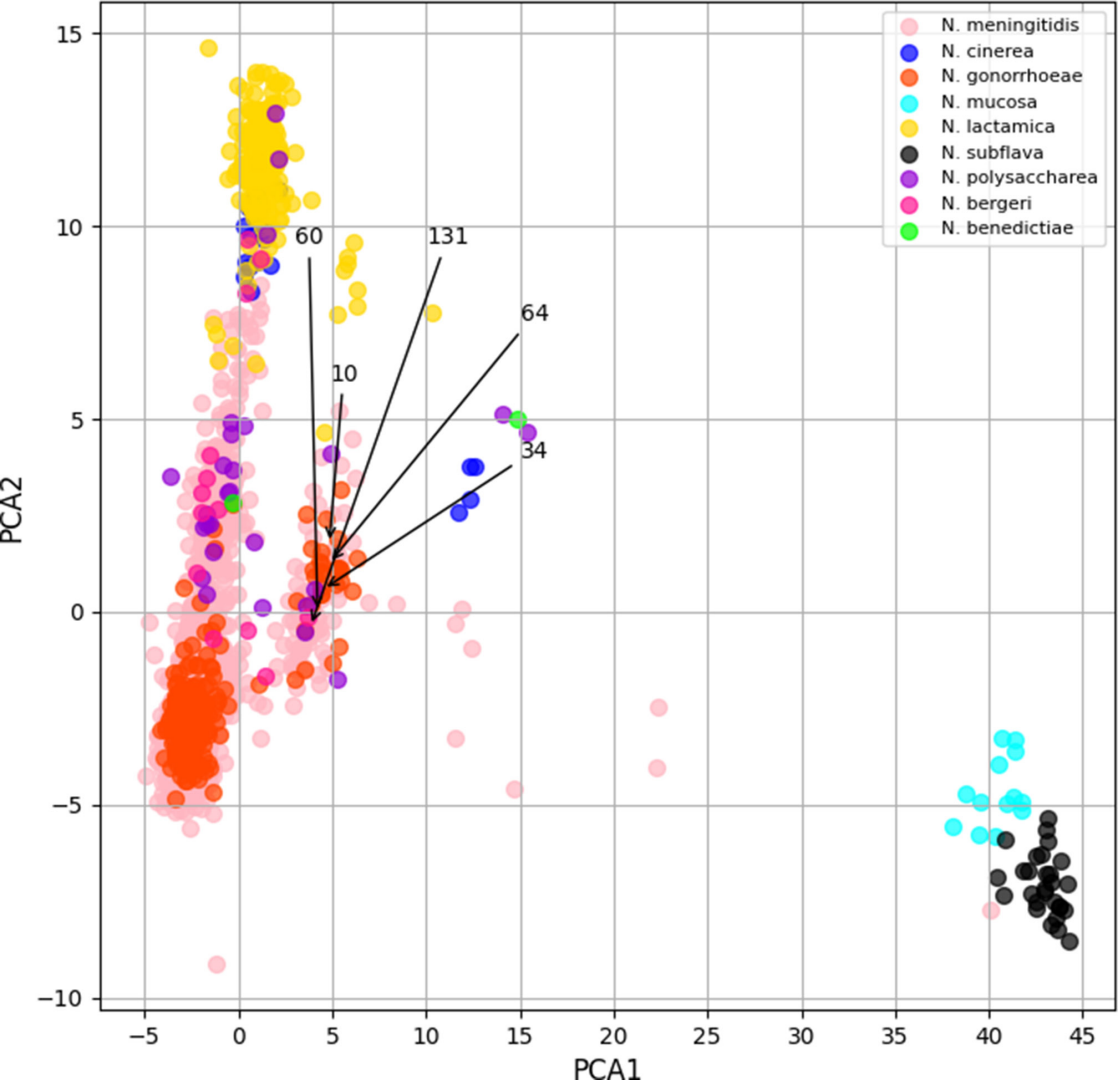
Principal component analyses of *penA* amino acid alignments. Amino acid alignments were converted to a numeric matrix and analysed by PCA using the sklearn.decomposition function in scikit-learn [32]. Four principal components were applied but two components were subsequently retained. Plots were visualized using the pyplot module in matplotlib and annotated by species with arrows denoting the location of particular mosaic types including *penA* 10, 34, 60, 64 and 131. Data points clustered by species consistent with phylogenetic analyses, but a smaller cluster can be observed which consists of *penA* alleles belonging to *N. gonorrhoeae, N. meningitidis, N. polysaccharea* and *N. lactamica* as well as mosaic *penA* types. There is considerable overlap between data points suggesting that this cluster of *penA* alleles represents a mosaic *penA* gene pool from which novel variants may arise.

Phylogenetic analysis via neighbour-joining trees indicated that the *Neisseria penA* gene consists of a population of alleles that group predominantly by species, contrasted with a sub-population of alleles that clustered across species boundaries, indicative of interspecies HGT (Fig. 2). This group included mosaic *penA* alleles. Subsequent phylogenetic and PCA analyses revealed that this cluster involved alleles found in 84 *N*. *meningitidis*, 31 *N*. *gonorrhoeae*, 28 *N*. *subflava*, 9 *N*. *lactamica*, 7 *N*. *polysaccharea*, 4 *N*. *cinerea,* and 1 *N*. *benedictiae, N. bergeri* and *N. blantyrii* isolates (Figs 4 and S1).

Most gonococcal mosaic *penA* alleles clustered together in this analysis, predominantly with other *N. gonorrhoeae* alleles, with the exception of some alleles belonging to mosaic types 35, 47, 63 and 131 which clustered with other *Neisseria* species including *N. meningitidis* and *N. polysaccharea* despite being found in *N. gonorrhoeae* isolates (Figs 4 and S1). These analyses allowed previously undescribed mosaic *penA* alleles belonging to *N. meningitidis* to be identified including NEIS1753_2308, a mosaic type 34 allele, and NEIS1753_471 and NEIS1753_861, both putative mosaic type 60.

### Shared alleles

NEIS1753 alleles 23, 166, 228, 266, 294 and 1240 were found in both *N. gonorrhoeae* and *N. meningitidis* (Table S1) consistent with the exchange by HGT of complete *penA* alleles, not only mosaic fragments. These alleles were more frequently found in gonococci with their presence in meningococci a rare event, ranging from one to three instances (Table S1). The exception was NEIS1753_266, which contains the *penA* mosaic 34 motif, as this was found in 50 meningococci of which 88 % belonged to the hyperinvasive clonal complex ST-11 and were globally distributed. The remaining isolates belonged to clonal complexes ST-174 (*n*=2), ST-254 (*n*=1) and ST-41/44 (*n*=1). NEIS1753_1240, an additional mosaic type 34 allele, was found in only one ST-11 meningococcus. NEIS1753_142 was identified in both *N. bergeri* and *N. polysaccharea*, and NEIS1753_908 in both *N. meningitidis* and *N. polysaccharea* (Table S1). Certain MLST sequence types were more commonly associated with shared *penA* alleles than others (Table S1).

The first instance of each of the shared alleles in this dataset are in gonococci. For example, the earliest NEIS1753_266 allele identified dates from 2001 and belongs to a gonococcus, while the first *N. meningitidis* NEIS1753_266 was not collected until 2013.

### Characterization of *penA* amino acid polymorphisms implicated in reduced susceptibility

Five amino acid substitutions in *penA* have been implicated in conferring reduced susceptibility: I312M, V316T, N513Y, G546S and more recently A502V [14, 16]. The position numbering used here follows that described by Ohnishi *et al*. [18], although the latter three polymorphisms have been described in other studies one residue earlier [16, 28]. This is due to the use of alignments which consider residue 346D an insertion; however, the presence of 346D is far more common than its absence in this dataset. In the 216 *N*. *gonorrhoeae* alleles identified here, the insertion of 346D was present in 68 % of alleles (147/216), and we therefore recommend that *penA* residues be described using the numbering system applied here (Fig. S2).

The prevalence of these polymorphisms, and which amino acid combinations were of highest incidence, was assessed. A concatenated sequence or motif consisting of these five amino acids was used to delineate the different combinations of polymorphisms found. For example, IVANG, representative of I312–V316–A502–N513–G546 (i.e. no substitutions, wild-type *penA* variant) or MTVYS representing M312–T316–V502–Y513–S546 (AMR-associated variant)(Fig. S2). A total of 20 different motifs were found (Table S2) with the wild-type, IVANG, found in the majority of *penA* alleles (1054/1700, 62 %) and across all *Neisseria* species except *N. mucosa, N. oralis* and *N. subflava* which all possessed the motif MTANG instead, with the amino acid substitutions I312M and V316T. This motif was found in 123/1700 (7 %) of isolates and in all species. The N513Y substitution was found in 449 alleles (449/1700, 26 %) and was identified in 12 of the 15 species examined. This substitution was frequently found in the IVAYG combination and in *N. cinerea* (4/26, 15 %), *N. lactamica* (124/194, 64 %), *N. polysaccharea* (7/28, 25 %), *N. meningitidis* (258/1173, 22 %) and *N. gonorrhoeae* (7/216, 3.2 %). Of the five amino acid substitutions investigated, G546S was the least common, identified in only 35/1700 (2 %) alleles, including alleles that were classed as mosaic type 34, 10 or 60.

The MTAYS motif, which is present in mosaic *penA* types 10, 34 and 60, occurred in only 28 *penA* alleles in our dataset (28/1700, 1.65 %), the majority of which belonged to *N. gonorrhoeae* isolates (23/28, 82 %). Four *N. meningitidis* isolates also possessed *penA* alleles with the MTAYS motif (alleles 1240, 2083, 2308 and 2408). As indicated previously, NEIS1753_1240 is an allele that is more commonly found in *N. gonorrhoeae*. NEIS1753_2083 clustered with a known *N. gonorrhoeae* mosaic *penA* type 35 while NEIS1753_2308 clustered with gonococcal *penA* mosaic variants including 10 and 34 (Fig. S1).

These MTAYS motif *penA* alleles were found in meningococci originating from the USA, China, the UK and Vietnam (NEIS1753 alleles 1240, 2083, 2308 and 2408 respectively). The Chinese isolate belonged to the hyperinvasive clonal complex ST-4821, the UK meningococcus from the ST-162 clonal complex and the Vietnamese isolate from ST-5571, which is not assigned to a clonal complex. The Vietnamese isolate was the only one associated with invasive disease, the other two originating from carriage. Motif MTAYS was found in one ‘*N. benedictiae’* isolate, OX40911 (NEIS1753_935). The species ‘*N. benedictiae’* was first described in Africa, but this particular isolate, OX40911, was obtained during a UK carriage study in 2015.

Although the MTAYS motif was present in all alleles belonging to mosaic types 10, 34 and 60, its presence there was not solely as a result of HGT into these alleles. While the substitutions I312M and V316T were both within the *N. subflava*-derived recombinant region defined by RDP5 – and 312M and 316T were present in all *N. subflava* alleles in our dataset – the substitutions N513Y and G546S were outside of the recombinant regions identified by RDP5 in *penA* type 34 alleles, with the furthest breakpoint at position 491. Furthermore, N513Y was only rarely present in *N. cinerea,* and never in *N. subflava,* while G456S was not found in any *N. subflava* or *N. cinerea* alleles in this dataset. Together, these findings suggest N513Y and G546S may have occurred in mosaic type 34 alleles *de novo,* rather than by HGT.

The substitution A502V, shown to increase ceftriaxone and cefixime minimum inhibitory concentrations (MICs), is a more recent addition to the list of *penA* polymorphisms implicated in AMR [14]. In our dataset this substitution was present in 43/1700 alleles (2.53 %). These alleles were found in either *N. gonorrhoeae* or *N. meningitidis*, but did not belong to the resistance-associated mosaic *penA* types 10, 34 or 60.

### Recombination analyses

A total of 333 unique recombination events were identified using a curated dataset consisting of 1061 alleles obtained from 15 *Neisseria* species. Nine species, comprising *N. bergeri, N. cinerea, N. gonorrhoeae, N. lactamica, N. maigaei, N. meningitidis, N. mucosa, N. polysaccharea* and *N. subflava,* were determined to act as minor parents in these events, in that *penA* alleles originating from these species were predicted to have contributed fragments in the generation of recombinant mosaic alleles. The most common species implicated was *N. meningitidis,* identified 911 times as a minor parent (911/2515 total minor parental sequences, 36 %), followed by *N. subflava*, identified 379 times (379/2515, 15 %), and *N. cinerea*, identified 376 times (376/2515, 15 %) (Fig. 4a). Note that each of the 2515 total minor parent sequences in our dataset was originally transferred into the *penA* gene as a result of one of the 333 unique recombination events RDP5 detected. These minor parental sequences are found in multiple alleles as they have spread by clonal expansion and been altered by further mutation or recombination.

The species implicated as minor parents in recombinant *penA* alleles differed between *N. gonorrhoeae* and *N. meningitidis. N. meningitidis* was the most common minor parent identified in *N. meningitidis* recombinants, indicative of frequent intraspecies recombination (Fig. 4b). The second most common minor parent in *N. meningitidis* recombinant *penA* alleles was *N. lactamica*. In contrast, the most commonly identified minor parent in *N. gonorrhoeae* was *N. subflava* followed by *N. cinerea* (Fig. 4c). Previous studies have implicated both of these species as contributing to the generation of mosaic *penA* types 10 and 34 in *N. gonorrhoeae* [10, 37, 38].

Although some of the *Neisseria* species identified as minor parents in *N. meningitidis* and *N. gonorrhoeae penA* alleles were the same, *N. gonorrhoeae* experienced recombination with fewer species – *N. bergeri*, *N. mucosa* and *N. polysaccharea* were absent. Furthermore, the incidence of recombination events was different. For example, a much higher incidence of *N. subflava* acting as a minor parent was observed in *N. gonorrhoeae* compared to *N. meningitidis* where intraspecies recombination was more evident. This suggests that either different opportunities for interspecies recombination exist for these two species or that different selective pressures affect which recombinant alleles become fixed in their populations.

The most common minor parent allele for recombinant gonococcal *penA* alleles was identified as *N. subflava* allele 301 (Table 2). Limited information is available on the isolate from which this allele originates (CCUG 24841). It was initially deposited in CCUG (https://www.ccug.se/), a culture collection, by J.-Y. Riou, of CIP, Paris, France, in 1989. In our dataset allele 301 is unique to this isolate with the most closely related allele belonging to *N. meningitidis* (allele 1464, 99.94 % sequence identity). Although the identification of alleles implicated in HGT is restricted to those present in the dataset used, these analyses show that *N. subflava penA* allele 301 is identified as a minor parent of *penA* type 34, a known resistance-conferring *penA* type. Indeed, mosaic type 34 alleles were consistently identified by RDP5 as having minor parent alleles from *N. subflava* and *N. cinerea*. Breakpoints for these recombination events were output as nucleotide position 909–1475 for the fragment from *N. subflava,* and from 564 to at least 892 for *N. cinerea*. A similar mosaic type implicated in resistance, type 10, was also identified as having *N. subflava* and *N. cinerea* as minor parents, although there was an additional recombinant region identified of unknown origin. NEIS1753_1548, belonging to mosaic type 60, was identified by RDP5 as having sequence derived from *N. subflava* and *N. lactamica*.

**Table 2.** Incidence of species as minor parent in recombination analyses across 1061 *penA* alleles Recombination analysis performed using RDP5 identified putative recombination events and the minor parental species in these events. Minor parents are alleles predicted to have contributed the smaller fraction of a recombinant allele. The most common minor parent across the analysis was *N. meningitidis. ‘*Unknown’ followed by a species indicates the program’s best guess where statistical support was not high enough. These were not included in further analysis.

Species	Count of incidence as minor parental sequence	%
*N. meningitidis*	911	36.22 %
*N. subflava*	379	15.07 %
*N. cinerea*	376	14.95 %
*N. lactamica*	344	13.68 %
Unknown (*N. meningitidis*)	313	12.45 %
Unknown (*N. lactamica*)	61	2.43 %
*N. bergeri*	55	2.19 %
*N. gonorrhoeae*	24	0.95 %
*N. mucosa*	19	0.76 %
Unknown (*N. cinerea*)	11	0.44 %
*N. polysaccharea*	10	0.40 %
Unknown (*N. mucosa*)	7	0.28 %
Unknown (*N. subflava*)	2	0.08 %
*N. maigaei*	1	0.04 %
Unknown (*N. gonorrhoeae*)	1	0.04 %
Unknown (*N. polysaccharea*)	1	0.04 %
**Grand total**	**2515**	

## Discussion

Mosaic forms of the *penA* gene in the pathogenic *Neisseria,* particularly *N. gonorrhoeae,* have been implicated in resistance to beta-lactam antibiotics since the 1990s, with commensal *Neisseria* frequently identified as the contributors of recombinant genetic material to these alleles via transformation [6–8]. Here, we undertook population-level analyses of the *penA* gene, examining its diversity in alleles spanning the genus *Neisseria*, and gaining insight into the origins of *penA* mosaic regions. Our results indicate that novel mosaic variants in *N. gonorrhoeae* originate in the first instance through HGT from sources of more divergent *penA* alleles, predominantly *N. subflava* and *N. cinerea*. These variants are subsequently repeatedly re-sampled between *Neisseria* species.

We investigated the *penA* gene across 15 *Neisseria* species, with our preliminary analysis revealing different levels of variation in *penA* across the genus. This suggests that differing evolutionary pressures are acting on the *penA* gene in different *Neisseria species*. For example, the lower *penA* diversity we observed in *N. gonorrhoeae* potentially represents positive selection for reduced susceptibility to antibiotics, resulting from increased selective pressure on the *penA* gene due to the frequent use of antibiotics to treat gonococcal infection [39]. Conversely, the higher *penA* diversity observed in the typically commensal *N. meningitidis* may reflect weaker selection pressure, alongside the generally higher genetic diversity found in the larger meningococcal population [40].

Our recombination analyses investigated these species-specific patterns further, finding that intraspecies recombination dominated in *N. meningitidis penA*, while interspecies HGT was more frequent in *N. gonorrhoeae* with the most common minor parent being *N. subflava*, followed by *N. cinerea*. These two commensal species have previously been identified as the source of recombinant DNA found in *penA* mosaic types 10 and 34 – types commonly recognized as the predominant cause of reduced susceptibility to ESCs in gonococci [8, 11, 18].

Research has shown that commensal *Neisseria* species such as these are developing reduced susceptibility to antimicrobials due to bystander selection [41, 42]. Ostensibly, this results in the emergence of resistance-associated substitutions within *penA* in these species, which can then cross species boundaries via HGT. This is consistent with the identification in this study of *penA* alleles in multiple commensal *Neisseria* species that contained resistance-associated polymorphisms. The co-localization of commensal *Neisseria* species with *N. gonorrhoeae* in the oropharynx during oral gonorrhoea enables the interspecies HGT of these motifs into the normally urogenital pathogen, conferring resistance to ESCs [43–46].

Our recombination analysis showed that the four commensal species identified as contributing to recombinant *N. gonorrhoeae penA* alleles, *N. subflava, N. cinerea, N. lactamica* and *N. meningitidis*, were those species that possessed the most highly diverse *penA* alleles (Table 1 and Fig. 1). This supports the theory that *N. gonorrhoeae* is sourcing *penA* sequences from these species as a more varied gene pool that facilitates the generation of novel *penA* alleles, acquiring resistance-associated polymorphisms in the process.

The broad dataset of *penA* alleles collated here enabled us to characterize the distribution of these polymorphisms across the genus *Neisseria*. The motifs associated with AMR were identified within multiple *Neisseria* species: evidence of the role of commensal *Neisseria* as a pool from which pathogenic *Neisseria* can source resistance determinants [43]. However, in our analysis we found that the polymorphisms 513Y and 546S were not yet associated with recombinant regions, instead appearing to have evolved *de novo* in current mosaic *penA* alleles, emphasizing the complex nature of resistance-associated changes in *penA*. The presence of these polymorphisms across the genus has implications for the use of multiplex PCR assays including nucleic amplification assays (NAATs) that target these sites to detect resistant gonococci, indicating that simultaneous species-specific identification assays should also be conducted to avoid false positive results [47, 48].

Early on in this study we detected identical *penA* alleles in more than one species, suggestive of interspecies HGT of complete *penA* alleles. Evidence of interspecies transfer of whole *penA* alleles was rarely observed, possibly as a consequence of epistatic effects that could lower the fitness of whole *penA* alleles transferred between species [16]. Six of the eight shared alleles are only shared between *N. gonorrhoeae* and *N. meningitidis*, which indicates that overall sequence identity might play a part; these species are more closely related to each other than to other *Neisseria* [49]. The distribution of shared alleles between these two species, with each allele more common in gonococci, is likely to be the result of the occasional spill-over of successful mosaic *N. gonorrhoeae penA* alleles into the *N. meningitidis* population through HGT. This is supported by the observation that the earliest incidences of each of these alleles are in gonococci. Furthermore, shared allele NEIS1753_266 was identified in meningococci belonging to the ST-11 clonal complex, a lineage associated with meningococcal urethritis, clonal complex 11 [50]. This suggests that NEIS1753_266 may have moved from gonococci to *N. meningitidis* through HGT facilitated by coexistence in the urogenital niche, an inference reinforced by the fact that this clade is known to possess an increased amount of recombinant DNA of gonococcal origins [51]. This horizontal spread of complete *penA* alleles has important implications for the dissemination of antibiotic resistance. Rather than mosaic regions being transferred into different genetic backgrounds, complete transfer across species boundaries may alter the pace at which resistance-inducing *penA* variants can spread within the genus.

In conclusion, these findings indicate that AMR-associated changes in the *Neisseria penA* gene are widespread across species boundaries. Bystander selection occurring in oral niche commensal *Neisseria* is likely to be partially responsible, combined with the increasing incidence of oral gonorrhoea providing improved opportunity for the HGT of these polymorphisms into gonococci. Therefore, in order to slow the development and spread of AMR in gonococci, the oral niche and the other *Neisseria* species that persist there must be considered carefully in both surveillance and treatment strategies. Although oral gonorrhoea presents little threat to health compared to its urogenital counterpart, it represents a key location for the emergence of resistance in *Neisseria*, evidenced by the identification of interspecies HGT with commensals in *penA* and other AMR determinants [5, 43, 52]. Enhanced surveillance and treatment in this niche could slow the acquisition of new mosaic *penA* alleles by *N. gonorrhoeae*, and hence represent an important step in preserving our last available treatments.

## Supplementary Data

Supplementary material 1
